# Tacrolimus for Remission Induction and Maintenance Therapy in Patients with Ulcerative Colitis: A Retrospective Evaluation Study

**DOI:** 10.1155/2016/5956316

**Published:** 2016-06-16

**Authors:** Ayumi Ito, Bunei Iizuka, Teppei Omori, Shinichi Nakamura, Katsutoshi Tokushige

**Affiliations:** Department of Gastroenterology, Tokyo Women's Medical University, Kawada-cho 8-1, shinjuku-ku, Tokyo 162-8666, Japan

## Abstract

*Background*. In this retrospective study, we compared the efficacy of tacrolimus (TAC) or prednisolone (PSL) for maintenance therapy in patients with ulcerative colitis (UC) at remission.* Methods*. The study patients were followed up for at least one year after induction of remission with either PSL (*n* = 55, between April 2004 and March 2014) or TAC (*n* = 40, between April 2009 and March 2014). The clinical features and relapse rates were compared in the two groups. Maintenance therapy in the TAC group included TAC alone, AZA alone, and TAC plus AZA.* Results*. The recurrence rates at 1500 days after remission were 61% and 46% for the PSL and TAC groups, respectively (*P* < 0.05). The recurrence rates at 600 days for TAC, AZA, and TAC + AZA maintenance groups were 24%, 49%, and 55%, respectively. Nephrotoxicity developed in 16 patients on TAC maintenance therapy.* Conclusions*. TAC monotherapy is a potential alternative especially for PSL nonresponders or those intolerant to AZA. However, patients on TAC therapy should be regularly monitored for adverse effects including nephrotoxicity.

## 1. Introduction

Ulcerative colitis (UC) is a chronic relapsing and remitting inflammatory bowel disease (IBD), which afflicts millions of individuals throughout the world with debilitating symptoms, impaired performance, and poor quality of life [1]. The mainstay of treatment for UC has been 5-aminosalicylic acid (5-ASA) preparations, dietary intervention, corticosteroids (e.g., prednisolone (PSL)), immunomodulator drugs such as azathioprine (AZA), and more recently anti-tumor necrosis factor- (TNF-) *α* biologics.

PSL is often used for severe cases, while 5-ASA is the first-line medication and AZA is often given as maintenance therapy to patients who have achieved remission with 5-ASA, corticosteroids, or another remission-inducing agent.

Although PSL is effective in the acute phase of UC with up to 85% response over the short term, it has far less long-term efficacy, with a reported one-year remission maintenance rate of 49% [2]. Furthermore, PSL has serious adverse side effects such as osteoporosis, cataract, and diabetes and has a negative impact on growth and development in young patients [3]. Indeed, routine management of UC requires administration of high doses of PSL for the treatment of acute severe flares and discontinuation of PSL after the induction of remission to minimize the side effects.

The development of the immunosuppressive agent, TAC, and novel anti-TNF-*α* antibody preparations in recent years has led to a decrease in the frequency of use of PSL or reduction in steroid dosage [4–15]. Indeed, in our hospital, TAC has shown good efficacy in induction of remission in patients with steroid-dependent or steroid-refractory UC. We often apply TAC for the treatment of patients with UC, who are otherwise unresponsive to conventional medications. The short-term remission rate with TAC has been as high as 80%. While AZA is commonly used for maintenance therapy after induction of remission, we are currently considering the use of TAC for maintenance of remission following induction therapy with TAC itself.

In this regard, the use of AZA for long-term maintenance therapy is associated with various side effects, such as allergic reactions, fever, nausea, and alopecia as well as more serious side effects like myelosuppression, infection, kidney and liver damage, skin cancer, and lymphoma [4, 5, 7, 8]. Accordingly, TAC should be considered for maintenance therapy especially if found effective and safe.

The aim of this retrospective study was to determine the efficacy and safety of TAC in patients with PSL-induced UC remission. Furthermore, we also determined the efficacy of TAC as maintenance therapy in subgroups of patients treated with TAC alone, patients who received TAC as induction therapy then switched to AZA as maintenance therapy, and patients on TAC with AZA added as maintenance therapy.

## 2. Materials and Methods

### 2.1. Study Design

**Figure 1:**
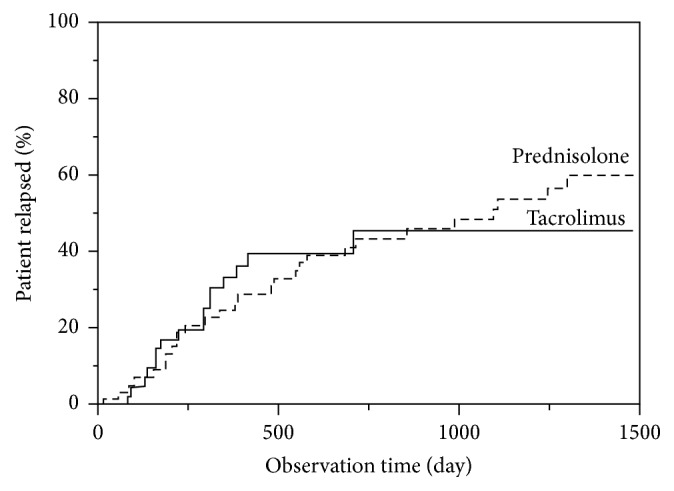
Cumulative relapse rate following remission induced by tacrolimus or by prednisolone in patients with ulcerative colitis.

In this retrospective study, 55 UC patients were followed up for at least one year after induction of UC remission with PSL at our hospital between April 2004 and March 2014 (PSL group), while 40 patients were followed up for at least one year after induction of remission with TAC between April 2009 and March 2014 (TAC group). The two groups (Figure 1) were compared with respect to the following variables: gender, age, disease duration, extent of UC, history of AZA therapy, pretreatment clinical activity index (CAI) according to Lichtiger et al. [10], duration of hospital stay (days), total dose of PSL until remission (mg), discontinuation of PSL after remission, pretreatment hemoglobin (Hb) and C-reactive protein (CRP) levels, the Mayo Endoscopic Scoring of Ulcerative Colitis [16], Ulcerative Colitis Endoscopic Index of Severity (UCEIS) [11], endoscopic activity index (EAI) [12], total dose of PSL during hospitalization, and discontinuation of PSL after induction of remission and latency to recurrence after induction of remission (Table 1).

**Figure 2:**
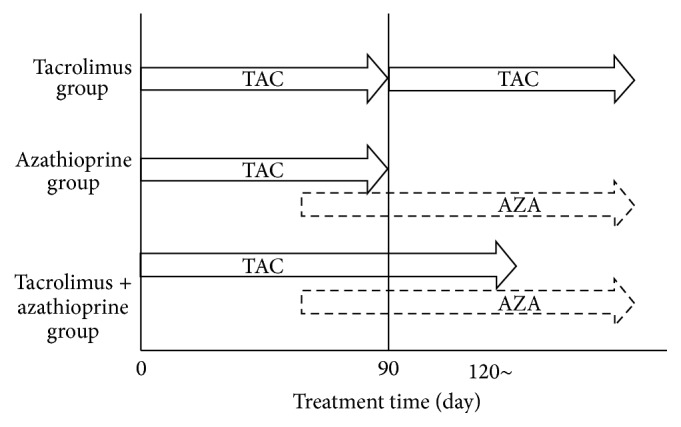
Duration of tacrolimus (TAC) therapy in the subgroups who received tacrolimus, azathioprine (AZA), or tacrolimus + AZA as maintenance therapy.

Among the 40 patients of the TAC group, 13 continued receiving TAC as maintenance therapy, 13 were switched to AZA as maintenance therapy, and 14 received TAC plus AZA as maintenance therapy (Table 2). All patients of the above three subgroups received the respective maintenance therapies for at least 90 days. In other words, 27 of the 40 patients received TAC for more than 90 days, while 13 received TAC for 90 days (Figure 2).

### 2.2. Doses of Prednisolone and Tacrolimus

PSL was administered orally at 0.5 to 1.0 mg/kg body weight/day, while TAC was given at 0.025 to 0.075 mg/kg body weight twice daily before breakfast and dinner. Patients of the TAC group were part of the PSL group but did not achieve remission due to more severe UC (Table 1). However, in both groups, PSL was to be tapered or discontinued after remission, whereas in patients with steroid-dependent UC, PSL was to be continued at a reduced dose. Blood samples were collected daily for measurement of TAC levels until the target blood concentration was reached. The TAC dose was adjusted to reach the target trough concentration of 10 to 15 ng/mL blood within two weeks of starting TAC remission induction therapy. Then, at 2 to 3 weeks after the TAC concentration was within the target range, the dose was adjusted again to reach a new lower target concentration of 5 to 10 ng/mL. The target concentration was also set at 5 to 10 ng/mL, when TAC was used for longer than 90 days as maintenance therapy. Remission was defined as CAI ≤4 after four weeks of TAC induction therapy. Likewise, recurrence was defined as the need to reinduce remission by increasing the dose of TAC to raise the trough blood level to ≥10 ng/mL, by intravenous PSL or by a biologic agent.

### 2.3. Statistical Analyses

Results are presented as either number of patients or mean ± standard deviation (SD) values. The Mann-Whitney test and the chi-square test were used for between-group comparisons. The recurrence rates were determined by the Kaplan-Meier estimator and compared by the log-rank test. The level of significance was set at *P* < 0.05. All statistical analyses were conducted using the software program called JMP Statistical Discovery (SAS, Version 11, SAS Institute, Japan).

## 3. Results

### 3.1. Comparison of Response to Prednisolone and Tacrolimus

There were no significant differences between the PSL group and the TAC group with respect to the gender ratio, age, UC severity level, past AZA therapy, pretreatment Hb, and CRP. However, there were significant differences between the two groups with respect to disease duration, pretreatment CAI, duration of hospitalization, Mayo score, UCEIS, EAI, and discontinuance of PSL after remission. Relapse after induction of remission was less frequent in the TAC group, with the recurrence rates for the PSL and TAC groups of 35% versus 40% at 500 days, 52% versus 45% at 1000 days, and 60% versus 46% at 1500 days (*P* < 0.05), respectively, indicating that, over longer observation period, more patients of the PSL group relapsed compared with the TAC group.

### 3.2. Remission Maintenance Outcome after Tacrolimus

**Figure 3:**
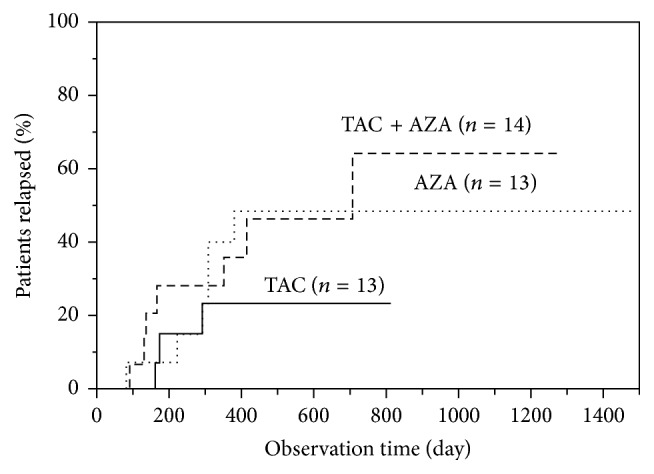
Cumulative relapse rate following remission induced by tacrolimus (TAC) followed by maintenance therapy with tacrolimus alone, azathioprine (AZA) alone, or tacrolimus plus AZA, in patients with ulcerative colitis.

There were no differences in the background factors (including a few patients who were on PSL) among the three TAC maintenance subgroups. The mean duration of TAC therapy was 402 ± 167 days in the TAC only subgroup, 87 ± 12 days in the AZA alone subgroup, and 240 ± 166 days in the TAC + AZA subgroup (Figure 2). The mean follow-up period after induction of remission with TAC was 698 ± 373, 1205 ± 349, and 806 ± 362 days in the TAC only, AZA alone, and the TAC + AZA subgroups, respectively. The recurrence rate at 300 days after induction of remission in the above subgroups was 28%, 32%, and 33%, respectively, and 24%, 49%, and 55% at 600 days. The above rates at 300 and 600 days were not significantly different (Figure 3). All patients were closely observed for TAC adverse effects, especially potential serious renal complications. Nephropathy was observed in 11 of the 40 cases, renal failure in 5 cases, and headache in one case. In the 5 patients who developed renal failure, the dose of TAC was reduced during treatment or discontinued altogether.

## 4. Discussion

Patients with active UC require immediate remission induction therapy to avoid further serious complications [13, 14]. Routine clinical practice includes the induction of remission followed by administration of another medication to maintain the remission [6, 7].

However, the use of an agent that induces remission and maintains remission has been rare [15]. In this study, we retrospectively evaluated the efficacy and safety of TAC for both induction and maintenance of remission. Importantly, the study included only patients who had achieved remission. The study design also allowed assessment of the efficacy of TAC as maintenance therapy, albeit in small subgroups of patients who had achieved remission with TAC.

Evaluation of induction of remission with PSL and TAC showed that disease duration pretreatment CAI, duration of hospitalization, Mayo score, UCEIS, EAI, and the proportion of patients on PSL were significantly different between the two groups. These differences reflected the fact that PSL was generally prescribed to induce remission in patients with moderately active UC or to patients who received treatment for the first time and readily responded to the medication [17]. In contrast, TAC was prescribed to induce remission in patients with more severe UC who had not responded well to PSL. In this regard, TAC is often used for treatment of patients with UC refractory to conventional medications [18]. In line with this conclusion, there was a significant difference between the two groups with respect to UC duration; PSL-naïve patients might have had the shortest disease duration. Another clinically relevant finding was the lower corticosteroid discontinuation rate after remission and higher recurrence rate in patients of the PSL group, with a significant difference in the latter at 1500 days between the two groups. These findings suggest that the total dose of PSL can be reduced and recurrence should be less likely when remission in UC patients is induced with TAC instead of PSL. However, there was no significant difference in the total dose of PSL during the hospitalization period.

Another clinically relevant feature of TAC is that it takes some time to achieve an effective trough concentration. Therefore, it may be appropriate to use PSL in combination with TAC during the acute phase, except in PSL refractory cases. Additionally, it is more difficult to use TAC than PSL because the blood concentration of TAC must be monitored to avoid serious adverse side effects [18–20].

Our analysis showed no significant differences in various background factors, including Hb and CRP levels before TAC administration, UCEIS, and EAI among the three TAC maintenance subgroups of TAC only, AZA alone, and TAC + AZA. Furthermore, no serious side effects were noted during maintenance therapy with TAC despite the fact that some groups [18–20] discouraged the long-term use of TAC. Among the 13 patients who showed maintenance of remission with TAC alone, 4 had previously used AZA but were refractory to that treatment or developed serious side effects to AZA. In some patients who develop side effects to AZA, AZA cannot be selected as maintenance therapy after induction of remission with TAC. It may be relevant to apply TAC for both remission induction and maintenance therapy in patients who cannot tolerate AZA [18–20].

Lower gastrointestinal endoscopic findings, including widespread ulcerations, deep ulcers, spontaneous mucosal bleeding, and marked mucosal edema, were common features in patients treated with TAC. In the presence of such findings, administration of TAC for 90 days is insufficient to achieve mucosal healing. Continued treatment with TAC should be considered, though compromise should be exercised in patients with adverse effects [18–20]. In this regard, complete mucosal healing is an important factor for achieving sustained remission, since patients with residual inflammation findings on endoscopy are vulnerable to recurrence [11–14]. Therefore, it is desirable to continue TAC for longer than 90 days to promote mucosal healing.

At our hospital, recurrence is less likely and maintenance of remission is achieved for a longer time in patients with mucosal healing, compared to those who achieved clinical remission without mucosal healing. Similar observations have been reported by Yamamoto et al. [21]. Currently, we perform lower gastrointestinal endoscopy after about 90 days of TAC therapy and then decide the next treatment option. After induction of remission following 90-day course TAC, UC recurrence is sometimes noted soon after switching maintenance therapy from TAC to AZA. Based on the ineffectiveness of AZA in some patients and the long lag time between administration and efficacy [22], remission can be reinduced in such patients with TAC and maintained by continuation of TAC therapy. It is important to maintain remission since drug therapy may not be effective for recurrent UC in some patients, who may otherwise require surgical intervention. Maintenance of remission is also important to minimize potential UC-related colorectal cancer, because long disease duration, UC extension beyond the left colon, chronic UC, and repeated recurrences are risk factors for colorectal dysplasia and cancer [22]. There is sufficient evidence that persistent inflammation contributes to the occurrence of cancer, whereas long-term maintenance of remission protects against cancer [13, 14]. Another clinically relevant issue is risk of infection during immunosuppressive therapy with more than two drugs [23].

Although the TAC group appeared to include patients with more severe UC, disease recurrence was more likely in the PSL group than in the TAC group following induction of remission. Furthermore, comparison of the outcome after induction of remission showed similar remission maintenance rates in the TAC alone, AZA alone, and TAC + AZA subgroups. This finding suggests that TAC monotherapy is a potentially viable option for maintenance therapy in UC patients who are intolerant to thiopurines. However, based on the known serious nephrotoxicity of long-term TAC therapy, there is need for a prospective randomized clinical trial of large number of patients to evaluate the efficacy and safety of maintenance therapy in patients with UC.

## Figures and Tables

**Table 1:** Baseline demographics of patients with ulcerative colitis (UC) who achieved remission with prednisolone (PSL) or tacrolimus (TAC) and were retrospectively reviewed.

Demography	PSL group (*n* = 55)	TAC group (*n* = 40)	*P* value
Male/female	36/19	22/18	NS
Age (years)	42.4 ± 17.5	43.7 ± 15.6	NS
Duration of UC (years)	5.6 ± 6.6	8.7 ± 1.3	<0.05
Extent of UC, pancolitis/left-sided colitis	48/6/1	24/16/0	NS
Past AZA therapy (yes/no)	35/20	29/11	NS
Clinical activity index (CAI)	11.8 ± 2.6	13.6 ± 2.8	<0.05
Duration of hospital stay (days)	32.5 ± 6.9	26 ± 10.2	<0.05
Total PSL until remission (mg)	702 ± 368	706 ± 354	NS
Discontinuance of PSL after remission (yes/no)	17/38	27/13	<0.05

Baseline Hb (g/dL)	12.3 ± 2.2	12.2 ± 2.5	NS
Baseline CRP (mg/dL)	3.8 ± 4.3	2.3 ± 3.3	NS

Mayo	2.7 ± 0.4	3.0 ± 0.0	<0.05
UCEIS	3.4 ± 1.9	5.1 ± 1.6	<0.05
EAI	12.3 ± 2.3	13.8 ± 2.4	<0.05

Data are mean ± SD or number of patients.

AZA, azathioprine; CRP, C-reactive protein; EAI, endoscopic activity index; Hb, hemoglobin; NS, not significant; UCEIS, Ulcerative Colitis Endoscopic Index of Severity.

**Table 2:** Clinical characteristics of patients with ulcerative colitis (UC) treated with tacrolimus (TAC) group who were randomly assigned to maintenance therapy with TAC, AZA, or TAC + AZA.

	TAC (*n* = 13)	AZA (*n* = 13)	TAC + AZA (*n* = 14)	*P* value
Male/female	9/4	7/6	6/8	NS
Age (years)	49.7 ± 16.4	42.4 ± 14.3	39.4 ± 15.3	NS
Duration of UC (years)	7.3 ± 7.7	11.3 ± 10	7.7 ± 8.2	NS
Follow-up time (days)	693 ± 359	1205 ± 349	806 ± 362	<0.05
Pancolitis/left-sided colitis	9/4	7/6	8/6	NS
Past AZA therapy (yes/no)	9/4	9/4	8/6	NS
Baseline CAI	13.7 ± 3.7	13.6 ± 3.0	13.5 ± 2.5	NS
Time to reach target blood TAC level (days)	3.6 ± 2.6	5.0 ± 2.5	3.9 ± 2.5	NS
TAC treatment time (days)	402 ± 167	87 ± 12	240 ± 166	—

Baseline Hb (g/dL)	10.8 ± 24	12.1 ± 2.3	13.3 ± 2.1	NS
Baseline CRP (mg/dL)	2.2 ± 2.7	3.7 ± 5.2	1.1 ± 0.7	NS

Mayo	3.0 ± 0.0	3.0 ± 0.0	3.0 ± 0.0	NS
UCEIS	6.3 ± 1.1	6.6 ± 1.8	6.4 ± 1.1	NS
EAI	13.5 ± 2.1	13.9 ± 1.6	13.6 ± 1.5	NS

## Abbreviations

5-ASA: 5-Aminosalicylic acid
AZA: Azathioprine
CAI: Clinical activity index
CRP: C-reactive protein
EAI: Endoscopic activity index
Hb: Hemoglobin
IBD: Inflammatory bowel disease
PSL: Prednisolone
SD: Standard deviation
TAC: Tacrolimus
TNF: Tumor necrosis factor
UC: Ulcerative colitis.
